# Potential Impact of Breathing Practice on Brain and Mental Health in Rural Adolescents With Psychiatric Symptoms: Protocol for a Single-Arm Pilot Feasibility Trial

**DOI:** 10.2196/91615

**Published:** 2026-11-11

**Authors:** Pravesh Sharma, Kaylynn Imsande, Danielle Boos, Nicholas L Bormann, Rowa Osman, Marin Nycklemoe, Seokbeen Lim, Stephan Arndt, Jana Kainerstorfer, Paul H Min

**Affiliations:** 1 Department of Psychiatry and Psychology, Mayo Clinic Health System, 1221 Whipple St., Eau Claire, WI, 54703, United States, 1 715 838 5369; 2 Central Wisconsin Campus, Medical College of Wisconsin, Wausau, WI, United States; 3 Department of Clinical Research, Mayo Clinic Health System, Eau Claire, WI, United States; 4 Department of Radiology, Mayo Clinic, Rochester, MN, United States; 5 Department of Biostatistics, University of Iowa, Iowa City, IA, United States; 6 Department of Psychiatry, University of Iowa, Iowa City, IA, United States; 7 Department of Biomedical Engineering, Carnegie Mellon University, Pittsburgh, PA, United States

**Keywords:** mental health, adolescent, rural, psychiatry, psychology

## Abstract

**Background:**

Mental health conditions (MHC), particularly depression and anxiety, are the leading contributors to youth disability globally. In the United States, there has been a steep increase in diagnosed MHC cases over the last decade. Adolescents in rural areas are disproportionately affected by the combination of limited access to mental health professionals and stigma around seeking care. Current treatment options, mainly psychotherapy and psychopharmacology, have shown modest effects. Prior research suggests associations between slow-paced deep breathing and autonomic function, cerebral perfusion, and stress regulation, supporting further investigation of structured breathing as a potential adjunctive approach for MHC management.

**Objective:**

This study aims to evaluate the feasibility, acceptability, implementation appropriateness, and participant satisfaction of a digitally supported, breathing-based intervention for adolescents with mild-to-moderate anxiety and depressive symptoms. The study will assess recruitment, retention, intervention engagement, usability, and the feasibility of collecting physiological and patient-reported outcome data to inform the design of a future efficacy trial.

**Methods:**

We aim to recruit approximately 40 adolescent patients receiving care through the Mayo Clinic Health System from rural communities in northwestern Wisconsin and southeastern Minnesota. Participants will complete a 4-month structured breathing program delivered entirely online, supported by a mobile app for guided instruction and practice tracking. All participants will attend 2 in-person visits (baseline and 4 mo) for functional near-infrared spectroscopy, as participants will already be on-site, the baseline and final study assessments will be completed at these same visits. A subsample (n=10) will additionally undergo magnetic resonance imaging at these visits.

**Results:**

This study was funded in October 2025, and data collection began in February 2026, with completion anticipated in late 2026. As of July 2026, 14 of the 40 (35%) planned participants have been enrolled. Analysis will follow completion of data collection, with results expected to be reported in early 2027.

**Conclusions:**

Adolescents with anxiety and depression are a particularly vulnerable group, often undertreated due to limited access to mental health care. The proposed breathing intervention is designed as an accessible and scalable approach that warrants feasibility evaluation in rural youth populations.

## Introduction

### Background

Mental health conditions (MHC), particularly depression and anxiety, are the leading contributors to youth disability globally. In the United States, over 5.3 million adolescents aged 12 to 17 (20.3%) had a current diagnosis of MHC in 2023, representing a 35% increase since 2016 [1]. Adolescents in rural areas often experience more severe emotional problems than their urban peers, partly due to limited access to mental health professionals, including psychiatrists, therapists, and other supports [2]. In addition, rural youth face stigma that is uniquely shaped by cultural norms, which can discourage help-seeking for MHC [3]. Untreated depression and anxiety may culminate in a state of chronic stress, a key contributor to altered brain development and impaired self-regulation, subsequently increasing the risk of negative outcomes such as substance use, academic struggles, and delinquency [4].

Adolescent anxiety and depression are currently treated using psychotherapy, psychopharmacology, or a combination of both. Selective serotonin reuptake inhibitors (SSRIs) are the most used pharmacotherapy to treat adolescent anxiety and depression; however, treatment response varies across individuals depending on factors such as diagnosis, symptom severity, and comorbidities, and many patients continue to experience residual symptoms despite treatment [5,6]. A 2022 meta-analysis of 34 randomized controlled trials (RCTs) involving 6161 children and adolescents with major depressive disorder reported a small effect size of 0.12 [5]. Similarly, a 2019 network meta-analysis of 22 RCTs including 2623 youth with generalized, social, and separation anxiety disorders found that SSRIs increased treatment response compared to placebo (odds ratio 4.6, 95% CI 3.3‐7.5), though many patients continued to experience residual symptoms [6]. Alternatively, adolescents and families may express a preference for nonpharmacological treatment options. In one survey, parents (N=143) favored counseling and physical activity over medication for managing adolescent depression [7]. Another study surveying adolescents who had received both medication and counseling found that 31% reported little to no benefit from medication, and 44% reported minimal benefit from counseling alone [8]. These challenges underscore the urgent need for accessible, low-barrier, self-paced strategies to augment existing treatments and support emotional and physiological regulation, particularly for adolescents in underserved areas with limited access to evidence-based treatment.

Slow, deep-paced breathing has emerged as a promising yet underutilized self-regulatory practice for individuals with MHC, with prior research suggesting associations with autonomic function, cerebral perfusion, and stress regulation [9-12]. Building on this literature, the present study focuses on the feasibility of implementing a structured breathing-based intervention and the feasibility of collecting brain physiological measures in adolescents with clinical anxiety and depression. Prior studies indicate that slow-paced breathing is associated with cardiovascular and neurophysiological processes relevant to stress and emotional regulation [9-14]. Consistent with this rationale, mindfulness-related processes have also been associated with more adaptive cognitive emotion regulation in adolescents, further supporting investigation of self-regulatory interventions in this population [15]. Structured breathing has also been associated with cerebrospinal fluid (CSF) flow, cerebral blood circulation, and brain metabolic processes [16,17].

Of particular interest is the prefrontal cortex, a brain region critically involved in emotional regulation, executive function, and autonomic control [18]. Altered structure and function of prefrontal circuits have been consistently implicated in both adolescent and adult anxiety and depression, particularly in relation to disrupted connectivity with limbic regions and reduced regulatory control over emotional and stress responses by the prefrontal cortex [19,20]. Prior work has shown that specific prefrontal subregions and their projection pathways may be disrupted in these conditions and may represent physiological systems relevant to evaluating breathing-based interventions [21,22]. Respiration-related fluctuations in blood-oxygen-level dependent signals and CSF dynamics, once considered physiological noise, are now detected via advanced functional magnetic resonance imaging (MRI), and are increasingly understood to reflect meaningful interactions between breathing and brain physiology [16,17,23,24].

Our preliminary work [16,17] suggests that respiration is associated with autonomic balance, cerebral circulation, and CSF movement. These physiological systems provide a mechanistic rationale for evaluating structured breathing interventions and can be feasibly assessed in adolescents to inform the design of future mechanistic and efficacy trials. Accordingly, the present study is explicitly designed to evaluate feasibility, acceptability, and implementation appropriateness of structured breathing intervention in adolescents with physiological and psychological measures examined descriptively rather than as efficacy end points.

Specifically, this study assesses the feasibility of collecting functional near-infrared spectroscopy (fNIRS) data in the full sample and the feasibility, tolerability, and completion rates of MRI in a subsample of 10 adolescents, without prespecifying neurobiological efficacy hypotheses. These imaging modalities are included to evaluate participant burden, data quality, and procedural viability in a rural adolescent population and to inform the design of future fully powered trials.

Given the increasing mental health burden among adolescents and persistent barriers to care in rural communities, self-regulatory practices, such as structured breathing, warrant feasibility evaluation as adjuncts to conventional treatment. To our knowledge, this is the first study to examine the feasibility, acceptability, and satisfaction with a structured breathing-based intervention while also evaluating the feasibility of multimodal physiological data collection (fNIRS and MRI) in rural adolescents with anxiety and depression.

This project is grounded in an equity-oriented approach to serving diverse and underserved populations. Adolescents with anxiety and depression are a particularly vulnerable group, often undertreated due to limited access to pediatric mental health specialists, extended wait times, and transportation barriers—challenges that disproportionately affect low-income families.

### Research Question

Can a structured breathing-based intervention for rural adolescents be feasibly implemented and perceived as acceptable, appropriate, and satisfactory by adolescents while also supporting adequate digital engagement through a mobile app and the feasible collection of physiological (fNIRS), psychological, and neuroimaging (MRI; subsample n=10) data to inform the design of future larger-scale trials?

### Hypothesis

The study hypothesizes that a structured breathing-based intervention will be feasible, acceptable, and appropriate for implementation among adolescents, as demonstrated by successful recruitment and retention and favorable scores on the acceptability of intervention measure (AIM), intervention appropriateness measure (IAM), and feasibility of intervention measure (FIM). It is further hypothesized that adolescents will demonstrate adequate engagement with the mobile app, reflected in adherence metrics, sustained use over the intervention period, and favorable usability ratings that inform barriers and facilitators to digital delivery.

Exploratorily, the study expects that the collection of fNIRS and psychological data will be operationally feasible with acceptable data completeness and tolerability. Descriptive summaries of these measures will be used solely to estimate variability, assess data quality, and inform the design of adequately powered future trials, without testing clinical or neurobiological efficacy or prespecifying directional effects.

### Specific Objectives

#### Primary Objective

To assess the feasibility, participant satisfaction, acceptability, and implementation appropriateness of a structured breathing-based intervention among adolescents. Feasibility will be evaluated based on recruitment rates, retention through the final assessment, and the completion of study procedures. Participant satisfaction will be assessed using satisfaction surveys, including quantitative ratings and open-ended responses. Acceptability and implementation appropriateness will be evaluated using standardized implementation outcome measures, including AIM, IAM, and FIM.

Preliminary feasibility data from our prior adult healthy control study support the plausibility of this approach. In that study, the recruitment rate exceeded 80% among eligible individuals approached, and participant compliance with the breathing protocol was approximately 85%, based on self-reported practice logs and session attendance. While these findings are not intended to predict outcomes in adolescents, they provide supportive evidence for the feasibility and acceptability of the breathing intervention framework.

#### Secondary Objective

To evaluate intervention adherence, usability, and engagement using metrics collected through the mobile app platform. App-based data will be used to characterize adherence patterns, sustained engagement over time, and user interaction with intervention components. These data will inform the identification of barriers and facilitators to digital engagement and guide refinements to intervention delivery and technological infrastructure for future larger-scale trials. Usability will be assessed using the System Usability Scale (SUS).

#### Exploratory Objective

To assess the feasibility of collecting physiological and psychological data in the context of a structured breathing-based intervention, including measures of cerebral oxygenation and local tissue hemodynamics using fNIRS in the full sample and MRI in a subsample of participants, alongside validated mental health measures. Exploratory analyses will focus on feasibility indicators such as data completeness, participant tolerance, and procedural viability. Descriptive summaries will be used to examine variability and estimate preliminary effect sizes to inform the design and power calculations of future adequately powered trials. The study is not powered to test clinical, physiological, or neurobiological effectiveness or efficacy hypotheses.

## Methods

### Reporting Framework

This protocol adheres to the SPIRIT (Standard Protocol Items: Recommendations for Interventional Trials) 2025 (Checklist 1) [25] statement for reporting clinical trial protocols; relevant guidance from the CONSORT (Consolidated Standards of Reporting Trials) 2010 extension for pilot and feasibility trials [26] was incorporated to align with the feasibility objectives of the study. The intervention is described according to the TIDieR (Template for Intervention Description and Replication) checklist (Checklist 2) [27] to facilitate complete reporting and reproducibility of the breathing intervention, including its rationale, procedures, instructor qualifications, mobile app functions, delivery format, practice progression, adherence monitoring, and permitted adaptations. Feasibility outcomes were informed by the framework proposed by Bowen et al [28]. and include assessments of acceptability, implementation, practicality, and demand using standardized implementation outcome measures (AIM, IAM, and FIM) [29].

### Progression Criteria

As a pilot feasibility study, this trial is not designed to evaluate intervention effectiveness but to determine whether a future definitive trial is warranted. Progression to a future RCT will be guided by predefined feasibility benchmarks, including recruitment of at least 75% of the planned sample within the recruitment period, retention of at least 80% through the 4-month follow-up, completion of physiological assessments (fNIRS and MRI, where applicable) in at least 80% of eligible participants, and mean AIM, IAM, and FIM scores of at least 4.0 on a 5-point scale. Adherence to the breathing intervention will also be summarized descriptively using app-based practice metrics. These benchmarks will guide interpretation of feasibility and identify aspects of the protocol requiring refinement before a future efficacy trial.

### Study Design and Setting

This is a single-arm, nonrandomized pilot feasibility study designed to evaluate the feasibility, acceptability, and implementation appropriateness of a structured breathing-based intervention for rural adolescents with mild to moderate anxiety or depression. The study will also assess the feasibility of collecting physiological and psychological measures to inform the design of future adequately powered trials. Approximately 40 participants will be recruited from Mayo Clinic Health System (MCHS) clinics serving rural communities in northwestern Wisconsin and southeastern Minnesota.

### Study Population

#### Eligibility Criteria

Participants must (1) be 12 to 17 years old, (2) have a clinical diagnosis of mild or moderate depressive disorder and/or an anxiety disorder, (3) be receiving care at one of 2 participating MCHS outpatient psychiatry clinics, and (4) must reside in rural areas as defined by Rural-Urban Commuting Area (RUCA) [30] codes (RUCA codes 4‐10). RUCA codes provide a standardized method for classifying rurality based on population density, urbanization, and commuting patterns, thereby capturing individuals living in small towns and outlying rural communities.

Participants must be able to read and understand English, and both parental or guardian consent and adolescent assent will be required. Adolescents with co-occurring mild to moderate alcohol or drug use problems will be eligible, provided these conditions do not require immediate specialized treatment or crisis intervention.

Exclusion criteria include current suicidal ideation or self-harm behavior requiring immediate clinical intervention, as well as severe psychiatric comorbidities such as psychotic disorders, bipolar disorder, major depressive disorder with psychotic features, severe substance use disorders, or eating disorders. Participants with severe cognitive impairment that would interfere with study participation will also be excluded. These criteria are intended to ensure participant safety and the feasibility of study procedures within a pilot implementation framework.

#### Recruitment

Potential participants will be identified through review of existing medical records at participating clinics and approached by members of the study team following approved recruitment procedures. Interested adolescents and their parents or guardians will receive detailed information about the study activities, including participation in the intervention and assessment requirements at each study time point, prior to providing informed consent and assent.

### Intervention

The breathing intervention is reported according to the TIDieR checklist to ensure complete and reproducible reporting of all intervention components. The intervention does not have an official or commercial program name and was developed specifically for this study as a structured breathing-exercise intervention. This study will use a fully online, structured breathing practice protocol to evaluate the feasibility and acceptability of delivering a breathing-based intervention to rural adolescents. The intervention combines guided virtual sessions with self-directed daily practice supported by a mobile app (Figure 1). The breathing program itself is delivered entirely online; this includes the one-time preprogram orientation with the instructor, all training, and all practice sessions, as well as the mobile app, which is used for both guided instruction and adherence tracking. The only in-person contact occurs at the 2 study visits (month 0 and month 4). All participants attend these visits for fNIRS data collection; because they are already on-site, the baseline and final study assessments are completed at the same visits. A subsample (n=10) additionally undergoes MRI during these 2 visits.

All participants will receive the same instructional materials and structured progression to maximize intervention fidelity. The breathing practice emphasizes soft, gentle movements intended to promote relaxation and awareness of breathing mechanics. Exercises are designed to support gradual familiarization with paced breathing and with postural engagement in a manner suitable for beginners.

Participants will follow a structured practice progression, beginning with 15-minute sessions and gradually increasing to 30 minutes and then 36 minutes per session over the course of the program. Participants will be encouraged to engage in self-practice at least 3 times per week. The program is designed to be accessible and adaptable, allowing participants to follow instructional video content and practice at their own pace. The mobile app serves both instructional and adherence-monitoring functions by providing standardized guided breathing sessions, practice reminders, and automated recording of session frequency, duration, and completion.

Breathing instructors will be informed only of each participant’s first name and age to facilitate instruction and safety but will not be provided with any additional study-related information. No direct identifiers or research data will be shared.

Monthly virtual check-ins will be conducted by the study team to collect survey data and to support participant engagement. These check-ins are intended to facilitate data collection and monitor feasibility-related outcomes rather than deliver therapeutic content.

Upon completion of the study, participants may elect to continue participating in the breathing program independently of the research study.

**Figure 1.**
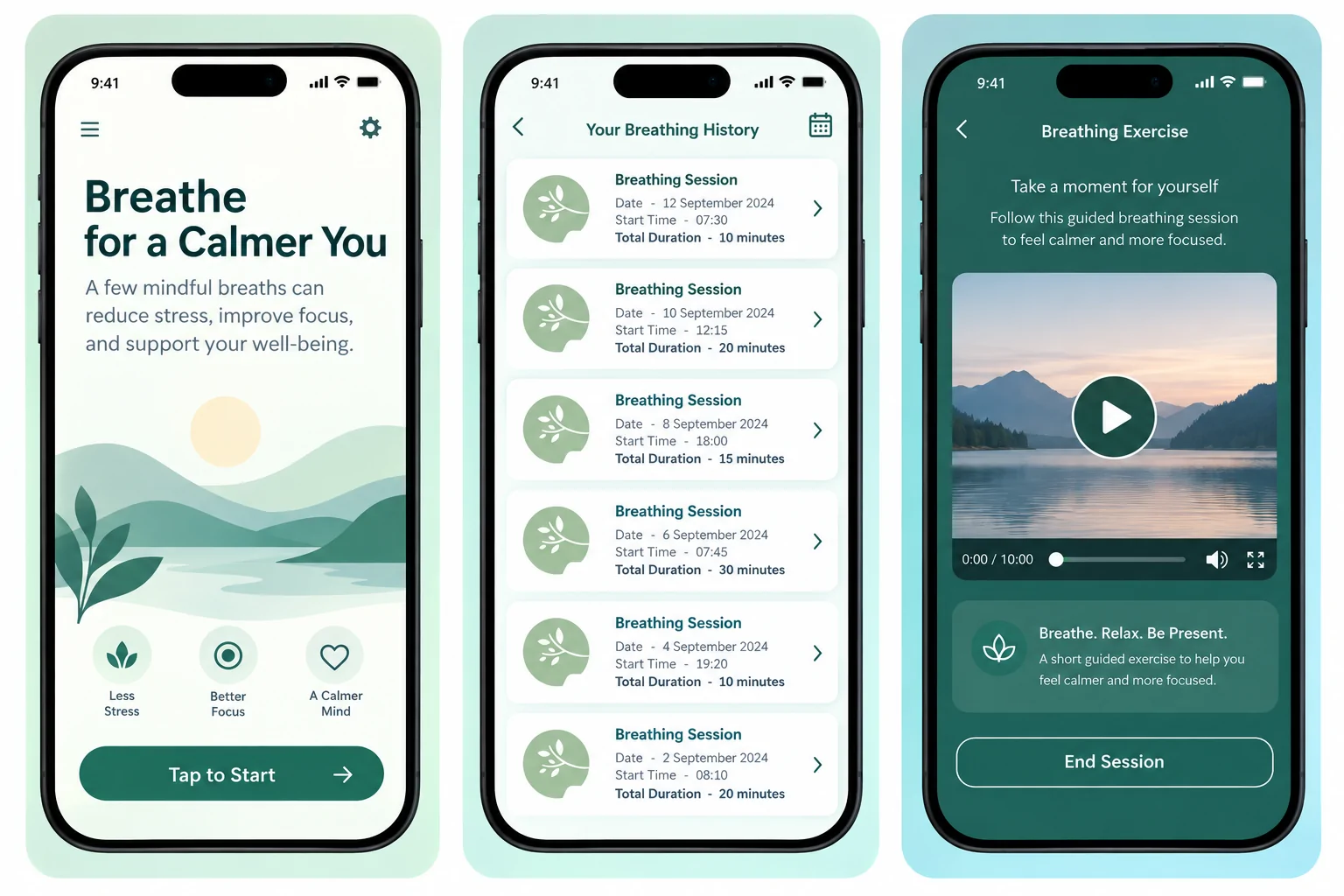
Mobile breathing practice app interface used in the study. It provides instructional videos, a session timer, and guided audio-visual cues. The app allows for personalization based on comfort and experience. The app includes features for tracking completed sessions, viewing session history, and receiving practice reminders. Usage data (eg, session type, duration, and timing) are logged and anonymized for research analysis.

### Data Management and Privacy

#### Mobile App Use

Mobile app data will be linked to participants through the study-specific identification numbers, and no protected health information will be collected or stored in the app. The mobile app is not considered an intervention component but serves solely as a tool for tracking engagement and adherence. The app records timestamps indicating when participants initiate and complete breathing practice sessions. Log data are transmitted daily via Wi-Fi or a cellular connection, depending on participants’ settings, and are stored using study identification numbers without direct identifiers.

The app is designed for 2-fold purposes: compliance tracking and instructional purposes. Participants can use the app to follow along and guide their practice. It is instructional in nature and helps to provide clear instructions on the different poses and postures, as well as how long one is expected to hold each posture. Each time a participant logs into the app to follow along with the instructional video, it will show the study team when and for how long the participant followed the video and completed the practice. This is how the app will be used as a compliance tracker as well.

#### Breathing Practice Sessions for Participants

To support safe participation while allowing flexibility in scheduling, the intervention includes a one-time initial instructional session followed by 2 ongoing formats: daily self-guided practice and optional weekly live online sessions. All sessions are designed to accommodate varying time commitments and learning preferences.

Participants are required to attend an initial online instructional session before beginning self-guided practice. During this session, participants receive instruction on the breathing protocol, guidance on progressing through the recommended practice schedule, and instruction on use of the mobile app.

1. Initial supervised online session: This one-time, real-time online session allows participants to join a live breathing practice led by the instructors, thereby supporting learning and familiarity with the breathing practices through real-time instruction.
2. Daily self-practice sessions: Daily self-guided practice follows a structured protocol with session durations of 15, 30, or 36 minutes, delivered via standardized online instructional videos on a video-hosting platform. The breathing practice mobile app is used to track session completion and adherence. Participants who experience discomfort or scheduling barriers may temporarily reduce practice frequency or duration before resuming the recommended progression while maintaining participation in study assessments.
3. Optional weekly online sessions: Optional weekly real-time online sessions allow participants to join live breathing practice sessions led by the instructors.

### Study Visits

Participants will complete 2 in-person study visits at baseline (day 0) and approximately 4 months (day 120) later. These visits will include the completion of study surveys and the collection of respiratory fNIRS data.

Following the baseline in-person visit, participants will attend an initial online breathing instruction session (approximately 60 min). This session will orient participants to the breathing practice protocol and provide instruction on the use of the mobile app. This session is instructional in nature and is intended to support safe and consistent participation rather than to deliver individualized therapeutic content. Participants will also complete 3 monthly online study visits (days 30, 60, and 90), during which study surveys and brief guided breathing check-ins will be completed. A scheduling window of up to 3 weeks before or after the planned study visits and survey time points will be permitted to minimize barriers to participation.

This flexibility applies to both in-person and online components of the study, including baseline and 4-month visits as well as monthly online visits. Participants are encouraged to communicate with the study team to identify convenient times for these monthly online visits. Participants are encouraged to maintain the practice on their own time and schedule, seeking a minimum of once per week for at least 30 minutes. The app acts as both an instructional device for the breathing practice and a way to track consistency and compliance.

### Ethical Considerations

This study was reviewed and approved by the Mayo Clinic Institutional Review Board (IRB number 25‐012922). All caregivers and participants will provide informed consent and assent, respectively, prior to participation, and procedures are designed to minimize risk and protect confidentiality. Participant safety will be safeguarded by trained clinical research coordinators. Data will be stored securely in REDCap, accessible only to authorized study personnel, and curated in accordance with Mayo Clinic policies and National Institutes of Health (NIH) data-sharing guidelines. Results will be disseminated through peer-reviewed publications and conference presentations. Deidentified datasets may be deposited in an appropriate research repository consistent with Mayo Clinic policy and funder requirements.

### Experimental Measures (Exploratory)

#### Overview and Feasibility Framework

Exploratory measures are included to assess the feasibility, tolerability, and data quality of collecting psychological and physiological data in the context of a structured breathing-based intervention. A stepwise feasibility approach will be used, with lower-burden measures implemented in the full sample and higher-burden neuroimaging procedures conducted in a subsample. Data from these measures will be used solely to evaluate procedural feasibility, estimate variability, and inform the design of future adequately powered trials; no clinical, physiological, or neurobiological efficacy hypotheses will be tested.

#### Survey Measures

Participants will complete a series of self-report surveys assessing mental health symptoms, well-being, and feasibility-related outcomes. Surveys include the General Health Questionnaire (GHQ), Patient Health Questionnaire-9 (PHQ-9), Generalized Anxiety Disorder-7 (GAD-7), Linear Analog Self-Assessment (LASA) for quality of life, Pittsburgh Sleep Quality Index (PSQI), Modified Fatigue Impact Scale (MFIS), Perceived Stress Scale (PSS), Self-Rated Abilities for Health Practices Scale (SRAHP), and an exit survey. Survey content may be simplified or omitted as necessary to reduce participant burden or eliminate redundancy.

Responses to the PHQ-9 and GAD-7 will be reviewed by a study psychiatrist (Drs Sharma and Bormann) after each study visit in accordance with the depression and anxiety management plan. Survey data will be collected and managed using REDCap.

#### Physiological Measures: Stepwise Feasibility Approach

Physiological data collection will follow a tiered feasibility strategy, with fNIRS implemented in the full sample as the primary physiological modality, followed by MRI in a smaller subsample to assess the feasibility of higher-burden neuroimaging procedures.

#### Respiratory and Basic Physiological Monitoring

At designated in-person study visits, intake and month 4, participants may undergo the collection of basic physiological measures to assess the feasibility and tolerability of the monitoring procedures. These measures may include respiratory belts, pulse oximetry or photoplethysmography (PPG), and electrocardiogram recordings using commercially available systems (eg, Biopac Systems, Inc; Obelab, Inc [31]). Specific modalities may be simplified or omitted if needed to reduce participant burden or address feasibility constraints.

#### fNIRS

fNIRS data will be collected using a portable, noninvasive system to assess the feasibility of measuring cerebral hemodynamic signals during study visits (Figure 2). The device estimates relative changes in oxygenated and deoxygenated hemoglobin concentrations in superficial cortical regions, including the prefrontal cortex, by emitting near-infrared light at 2 wavelengths (approximately 780 nm and 850 nm) and detecting wavelength-specific changes in light absorption (Figure 3).

fNIRS data will be used solely for feasibility-related purposes, including evaluation of signal stability, motion tolerance, participant comfort, and completeness of data acquisition. Data will be summarized descriptively to estimate variability and inform protocol refinement and sample size considerations for future studies. No mechanistic, physiological, or clinical efficacy hypotheses will be tested using fNIRS data in this study.

**Figure 2.**
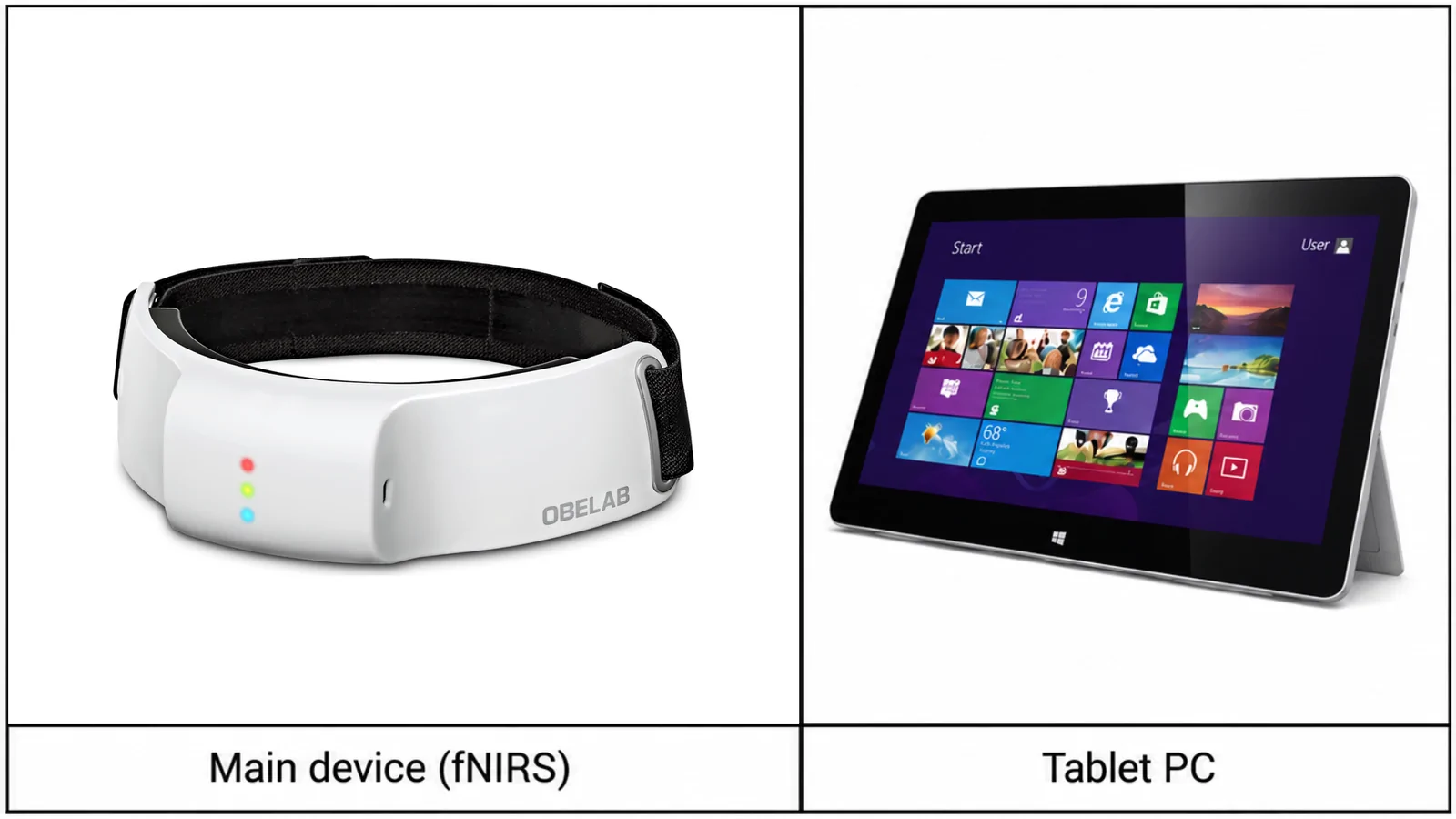
Functional near-infrared spectroscopy (fNIRS) device system. Image used with permission from OBELAB Inc.

**Figure 3.**
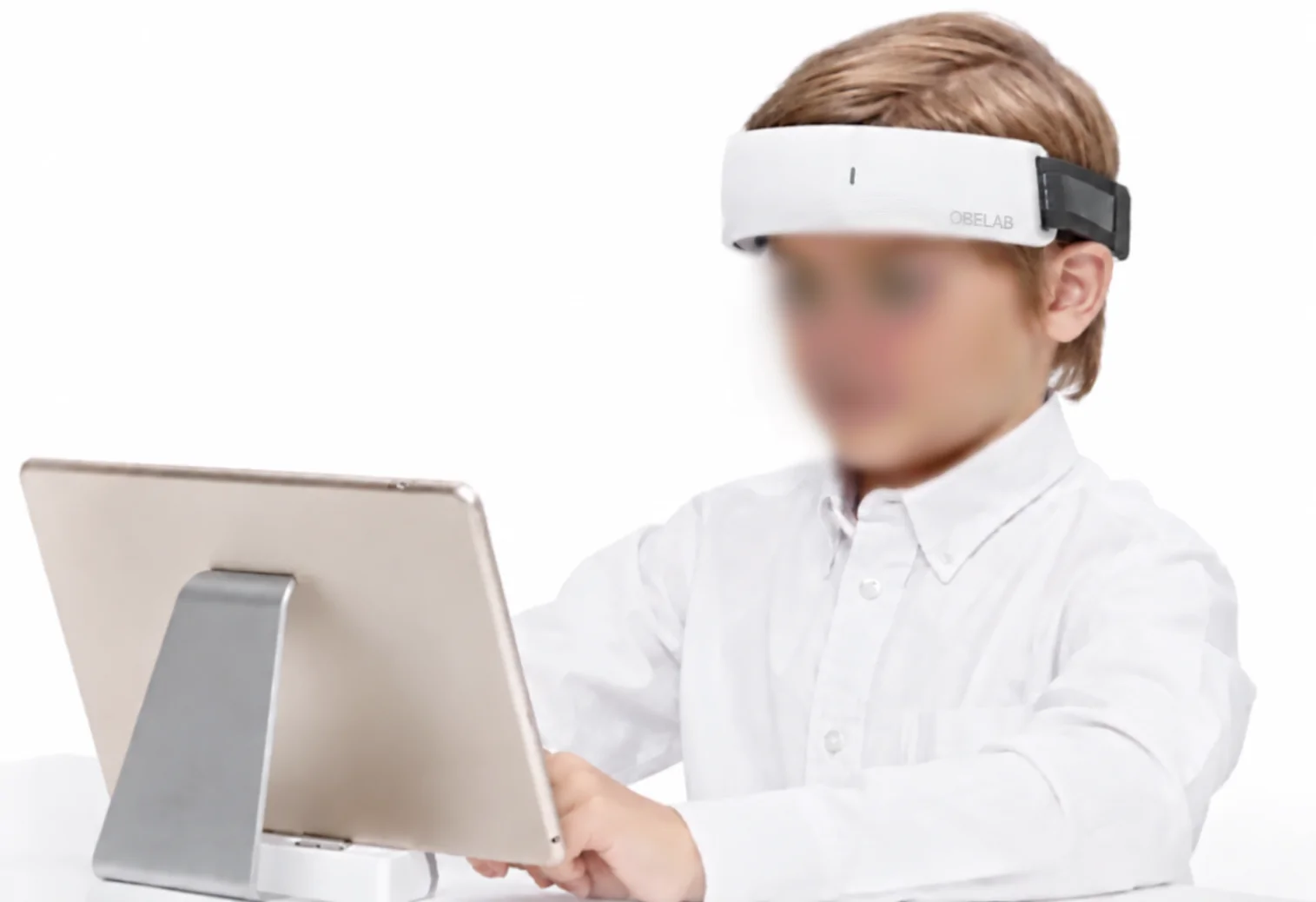
Functional near-infrared spectroscopy device worn on the head. Image used with permission from OBELAB Inc.

### MRI

A subset of participants (n=10) will additionally undergo MRI scanning to assess the feasibility, tolerability, and logistical requirements of incorporating MRI procedures into future larger-scale trials. MRI acquisition will occur after completion of fNIRS assessments and will be conducted using a 3T MRI scanner at MCHS–Eau Claire during after-hours, with support from 2 trained MRI technologists.

The MRI protocol may include phase-contrast MRI for flow-related measures and echo-planar imaging, followed by T1-weighted and T2-weighted anatomical imaging. For T2 imaging, both brain and abdominal scans will be acquired, with the abdominal scan included to assess the feasibility of capturing respiratory-related anatomical movement. MRI-compatible physiological monitoring (eg, PPG and respiratory belts) will be used to evaluate the feasibility of concurrent physiological data acquisition within the scanner environment.

A head-coil mirror and screen-based cue presentation will be used to display breathing-related prompts during scanning. If participant fatigue, discomfort, excessive motion, or sleep occurs, the scan sequences may be abbreviated, simplified, or discontinued. Scan repetition will be limited by feasibility considerations rather than data optimization. Total scan time will not exceed 70 minutes.

All MRI sequences will be conducted under FDA nonsignificant risk guidelines, as verified by a magnetic resonance physicist. The imaging protocol may be modified within these guidelines to accommodate feasibility, safety, or participant tolerance considerations.

MRI data will be used exclusively to evaluate feasibility parameters, including recruitment into the MRI subsample, scan completion rates, participant tolerance, motion-related data quality, and procedural logistics. Data will be summarized descriptively to inform the design of future adequately powered trials. No neural, physiological, or behavioral efficacy hypotheses will be tested with MRI data in this study.

### Adherence and Compliance

Our study includes a detailed adherence and compliance training program designed for the 4-month duration of the study.

1. Participant engagement: Participants will receive a report on their activity and will be asked to confirm whether there are any errors or updates needed. If a participant does not communicate with the study team for 5 days regarding their daily practice, the study team will send a reminder via their preferred contact method (eg, text message, telephone call, or email). This ensures ongoing engagement and addresses any participant concerns. For the breathing group, any feedback from the instructors will be relayed to the participants, and vice versa.
2. Emphasis on honest reporting: We encourage participants to report on their practice habits honestly and without any pressure. Self-practice is not mandatory, and there is no need to overcommit. The most important aspect of this study is that participants provide genuine feedback on their actual practice. The study seeks to understand natural compliance patterns, and we value honest, pressure-free reporting over strict adherence to practice frequency.

### Potential Adverse Events and Reporting Procedures

Potential adverse events (AEs) related to breathing practice, which involves light physical exercise, are generally minor and infrequent. Participants may experience dizziness, lightheadedness, shortness of breath, or headaches, particularly if they are new to breathing techniques or overexert themselves. Some individuals may report mild discomfort in the chest, muscle strain, joint discomfort, sleep problems, or increased anxiety during practice. Rarely, hyperventilation can occur, leading to tingling in the extremities or fainting. It is important to note that these events are typically transient and resolve quickly without lasting effects. Proper instruction and gradual introduction to breathing techniques can minimize the occurrence of these AEs. Participants must report any AEs to the study coordinator immediately and seek medical attention if necessary. In the S1_GHQ survey, we include a section to evaluate potential AEs of breathing practice.

### Depression Management Plan

The depression questionnaire (PHQ-9) responses are screened real-time by study coordinators. Any PHQ-9 total score of greater than 5 or any positive (ie, nonzero) response to the suicidal thoughts question (9) will be addressed promptly by the study coordinator by communicating with the study clinicians. Clinical psychiatric procedures for assessing and managing depression will be followed by the study physician psychiatrist (PS and NB).

### Renumeration

Participants will receive US $50 for each in-person visit completed at baseline (0 d) and at 4 months (120 d). Additionally, during the monthly online survey and breathing-practice check-ins at 1 month (30 d), 2 months (60 d), and 3 months (90 d), participants will receive US $20 for each completed online survey. Participants who complete all study activities may receive up to US $160 in total compensation.

### Outcomes and Measures

#### Primary Outcomes

The primary outcomes of this study focus on implementation feasibility and acceptability and encompass three domains: (1) acceptability, appropriateness, and feasibility of the breathing-based intervention; (2) recruitment and retention feasibility; and (3) participant satisfaction.

#### Acceptability, Appropriateness, and Feasibility of the Intervention

Acceptability, appropriateness, and feasibility of the breathing-based intervention will be assessed using standardized implementation outcome measures, including AIM, IAM, and FIM.

#### Recruitment and Retention Feasibility

Recruitment and retention feasibility will be evaluated by calculating the proportion of eligible adolescents who enroll in the study and the proportion of enrolled participants who are retained through the final study assessment.

#### Participant Satisfaction

Participant satisfaction will be assessed using satisfaction surveys, including quantitative ratings, open-ended survey responses, and the study exit survey.

#### Secondary Outcomes

Secondary outcomes focus on intervention engagement and digital delivery feasibility and include digital usability, engagement with the mobile app, adherence to the breathing practice, and caregiver-reported observations.

#### Digital Usability and Engagement

Digital usability and engagement will be assessed using app usability ratings and in-app analytics, including frequency of logins, session duration, and feature use.

#### Adherence to Breathing Practice and Intervention Engagement

Adherence and engagement will be assessed using breathing session attendance records, app-based adherence metrics, and completion rates of assigned breathing practice sessions.

#### Caregiver-Reported Observations

Caregiver-reported observations of adolescent mood, behavior, emotional regulation, and engagement with care will be collected via caregiver-completed surveys. These data are intended to provide contextual information regarding feasibility and perceived relevance of the intervention rather than to assess clinical effectiveness.

#### Exploratory Outcomes

Exploratory outcomes are included to assess the feasibility of collecting physiological and psychological data and to estimate variability to inform future trials. These outcomes are not intended to test efficacy or effectiveness.

#### Cerebral Hemodynamics (fNIRS)

The feasibility of assessing cerebral hemodynamic signals will be evaluated using fNIRS-derived estimates of relative changes in oxygenated and deoxygenated hemoglobin. Analyses will focus on data completeness, signal quality, and participant tolerance.

#### MRI (Subsample of 10)

In a subset of participants (n=10), the feasibility of MRI data acquisition will be assessed using structural and functional MRI sequences as previously described. Outcomes will include scan completion rates, participant tolerance, and data quality. MRI data will be summarized descriptively and will not be used to test neural or clinical hypotheses.

#### Emotional and Behavioral Well-Being Measures

The feasibility of collecting validated self-report measures of emotional and behavioral well-being will be assessed using the PHQ-9, GAD-7, GHQ, LASA, PSQI, MFIS, PSS, and SRAHP.

#### Descriptive Trends in Mental Health Measures

Descriptive pre-post summaries of validated psychological measures will be examined to estimate variability and inform power calculations for future trials. The study is not powered to test hypotheses or evaluate intervention effectiveness.

#### Data Analysis Plan

All primary and secondary outcomes will be summarized using descriptive statistics, including means, SDs, medians, proportions, and 95% CIs, as appropriate. Because this is a pilot feasibility study, the analytic focus is on estimation, variability, and data completeness, rather than hypothesis testing or formal statistical inference. Simple data visualizations (eg, pre-post plots, individual change trajectories, and distributional summaries) will be used to illustrate patterns and heterogeneity of responses. These summaries will inform the selection and prioritization of outcomes, including implementation, behavioral, and physiological measures, for future larger-scale trials.

For exploratory physiological and neuroimaging data, including fNIRS measures in the full sample and MRI data in a subsample of participants, analyses will focus on within-person descriptive changes from baseline to postintervention. Physiological recordings will be processed with standard preprocessing procedures to assess data quality, completeness, and signal stability. Summaries will characterize general patterns in cerebral oxygenation and basic physiological responses during study visits. These analyses are intended solely to evaluate the feasibility and interpretability of data collection procedures and to determine whether the observed variability suggests potential utility for inclusion in future studies; no efficacy or mechanistic hypotheses will be tested.

Qualitative feedback obtained from participants through open-ended survey responses will be analyzed using a brief thematic analysis approach. Two reviewers will independently review responses and identify common themes related to perceived benefits, challenges, acceptability, and overall impressions of the intervention. Any discrepancies will be resolved through discussion. Qualitative findings will be used to contextualize quantitative feasibility outcomes and to inform the refinement of the intervention and study procedures.

## Results

This study was funded in October 2025. Enrollment began in February 2026 and remains ongoing; as of manuscript submission (July 2026), 14 of 40 (35%) planned participants have been enrolled. Data collection is expected to conclude in late 2026. No interim analyses have been conducted; formal data analysis will commence after final data collection, with results anticipated for dissemination in spring 2027.

## Discussion

### Principal Findings

This pilot feasibility study is designed to determine whether a structured, app-supported deep-breathing intervention and multimodal physiological assessment protocol can be successfully implemented among rural adolescents with anxiety and depressive symptoms. We anticipate that the study will generate feasibility data regarding recruitment, retention, intervention acceptability, adherence, and the feasibility of collecting high-quality physiological and neuroimaging data. Rather than testing treatment efficacy, the primary contribution of this study will be to determine whether the intervention and study procedures are feasible and to generate estimates necessary for the design of a future adequately powered clinical trial. The results of this study will provide summaries of key feasibility outcomes and physiological and psychological data to inform the design and statistical planning of a subsequent fully powered trial. Accordingly, analyses will focus on recruitment and retention, intervention acceptability and participant satisfaction, adherence and digital engagement, and the data completeness, tolerability, and quality of multimodal data collection (fNIRS in the full sample and MRI in a subsample), rather than hypothesis testing. Outcomes will be summarized using descriptive statistics (means, SDs, medians, proportions, and 95% CIs, as appropriate). These findings will be used to refine study procedures, optimize assessment timing, and inform effect-size assumptions and sample-size calculations for future trials.

Although breathing-based interventions have been conducted to reduce anxiety, improve stress regulation, and enhance emotional well-being in adults [32,33], relatively few studies have examined their feasibility in rural adolescents, particularly using objective physiological measures alongside patient-reported outcomes. Furthermore, previous studies have rarely integrated portable neurophysiological assessments such as fNIRS. This study therefore addresses an important gap by providing feasibility data to inform the design of future effectiveness trials.

This project addresses two main priorities: (1) reducing health disparities and (2) enhancing population-based and value-based care in rural communities by evaluating the feasibility and acceptability of a low-burden, scalable breathing-based intervention for adolescents with anxiety and depression.

A major strength of the study is its integration of behavioral, psychological, and objective physiological measures while leveraging digital technology to support intervention delivery and adherence in a rural population. The inclusion of both portable neuroimaging (fNIRS) and MRI will also provide valuable information regarding the feasibility of collecting multimodal mechanistic data in this setting.

If feasibility benchmarks are achieved, future studies will expand recruitment across multiple rural health systems to generate a larger dataset and subsequently include suburban and urban settings to evaluate implementation across diverse populations and improve generalizability. Given the limited evidence on structured breathing interventions for adolescents living in resource-constrained rural communities, this work may provide the foundation for a future adequately powered multicenter RCT. Ultimately, this research may help determine whether a structured deep-breathing intervention can be feasibly implemented as a practical, low-burden adjunct to conventional care among rural adolescents.

As this is a pilot feasibility study, several limitations should be acknowledged. The single-arm design, relatively small sample size, and absence of a control group preclude conclusions regarding clinical effectiveness or neurobiological mechanisms. In addition, recruitment from rural outpatient psychiatry clinics within a single health system may limit the generalizability of the findings. The primary purpose of this study is to evaluate study procedures, participant engagement, and data collection processes that will inform the design of future adequately powered trials.

Study findings will be disseminated through presentations at regional and national scientific conferences, publications in peer-reviewed journals, and communication with participating clinical sites, community stakeholders, and participating families. The findings will also be used to guide the design and implementation of future multisite clinical trials evaluating structured breathing interventions for adolescent mental health.

### Conclusions

This pilot study will evaluate the feasibility and acceptability of a structured breathing-based intervention for rural adolescents with anxiety and depression. Findings on recruitment, participant retention, adherence, intervention acceptability and satisfaction, and data completeness will inform the design of future adequately powered RCTs evaluating the clinical effectiveness of this intervention.

## Supplementary material

10.2196/91615 Checklist 1 SPIRIT checklist.

10.2196/91615 Checklist 2 TIDieR checklist.

## Abbreviations

AE: adverse event
AIM: acceptability of intervention measure
CONSORT: Consolidated Standards of Reporting Trials
CSF: cerebrospinal fluid
FIM: feasibility of intervention measure
fNIRS: functional near-infrared spectroscopy
GAD-7: Generalized Anxiety Disorder-7
GHQ: General Health Questionnaire
IAM: intervention appropriateness measure
LASA: Linear Analog Self-Assessment
MCHS: Mayo Clinic Health System
MFIS: Modified Fatigue Impact Scale
MHC: mental health conditions
MRI: magnetic resonance imaging
NIH: National Institutes of Health
PHQ-9: Patient Health Questionnaire-9
PPG: photoplethysmography
PSQI: Pittsburgh Sleep Quality Index
PSS: Perceived Stress Scale
RCT: randomized controlled trial
RUCA: Rural-Urban Commuting Area
SPIRIT: Standard Protocol Items: Recommendations for Interventional Trials
SRAHP: Self-Rated Abilities for Health Practices Scale
SSRI: selective serotonin reuptake inhibitor
SUS: System Usability Scale
TIDieR: Template for Intervention Description and Replication
